# Over-prescription of short-acting β_2_-agonists and asthma management in the Gulf region: a multicountry observational study

**DOI:** 10.1186/s40733-022-00085-5

**Published:** 2022-07-07

**Authors:** Ashraf Alzaabi, Nasser Al Busaidi, Rohit Pradhan, Fathelrahman Shandy, Naseem Ibrahim, Moulham Ashtar, Khaled Khudadah, Khaled Hegazy, Mohamed Samir, Mohamed Negm, Hisham Farouk, Arwa Al Khalidi, Maarten Beekman

**Affiliations:** 1 Pulmonology Department, Zayed Military Hospital, Abu Dhabi, United Arab Emirates; 2 Pulmonology Medicine Department, Royal Hospital, Muscat, Sultanate of Oman; 3 Pulmonology Medicine Department, Rashid Hospital, Dubai Health Authority, Dubai, United Arab Emirates; 4 Primary Health Care Department, Emirates Health Services Establishment, Dubai, United Arab Emirates; 5 Health Affairs Department, Primary Health Care Services Sector, Dubai Health Authority, Dubai, United Arab Emirates; 6 Department of Family Medicine, Ahmadi Hospital, Al Ahmadi, Kuwait; 7 Sabah El-Salem Primary Healthcare Centre, Sabah Al-Salem, Kuwait; 8 Al-Rumaithiya Primary Healthcare Centre, Al-Rumaithiya, Kuwait; 9 Al Adan Hospital, Hadiya, Kuwait; 10 Medical Affairs Department, AstraZeneca, Dubai, United Arab Emirates; 11 AstraZeneca, The Hague, The Netherlands

**Keywords:** Asthma, Exacerbations, Gulf region, Over-prescription, SABINA, Short-acting β_2_-agonists

## Abstract

**Background:**

The overuse of short-acting β_2_-agonists (SABA) is associated with poor asthma control. However, data on SABA use in the Gulf region are limited. Herein, we describe SABA prescription practices and clinical outcomes in patients with asthma from the Gulf cohort of the SABA use IN Asthma (SABINA) III study.

**Methods:**

In this cross-sectional study conducted at 16 sites across Kuwait, Oman, and the United Arab Emirates, eligible patients (aged ≥ 12 years) with asthma were classified based on investigator-defined disease severity guided by the 2017 Global Initiative for Asthma report and by practice type, i.e., respiratory specialist or primary care physician. Data on demographics, disease characteristics, and prescribed asthma treatments, including SABA, in the 12 months prior to a single, prospective, study visit were transcribed onto electronic case report forms (eCRFs). All analyses were descriptive in nature. Continuous variables were summarized by the number of non-missing values, given as mean (standard deviation [SD]) and median (range). Categorical variables were summarized by frequency counts and percentages.

**Results:**

This study analyzed data from 301 patients with asthma, 54.5% of whom were treated by respiratory specialists. Most patients were female (61.8%), with a mean age of 43.9 years, and 84.4% were classified with moderate-to-severe disease, with a mean (SD) asthma duration of 14.8 (10.8) years. Asthma was partly controlled or uncontrolled in 51.2% of patients, with 41.9% experiencing ≥ 1 severe exacerbation in the 12 months preceding their study visit. Overall, 58.5% of patients were prescribed ≥ 3 SABA canisters, 19.3% were prescribed ≥ 10 canisters, and 13.3% purchased SABA over-the-counter (OTC) in the 12 months before the study visit. Most patients who purchased OTC SABA (92.5%) also received SABA prescriptions. Inhaled corticosteroid/long-acting β_2_-agonist combinations and oral corticosteroid bursts were prescribed to 87.7% and 22.6% of patients, respectively.

**Conclusions:**

SABA over-prescription was highly prevalent in the Gulf region, compounded by purchases of nonprescription SABA and suboptimal asthma-related outcomes. Increased awareness among policymakers and healthcare practitioners is needed to ensure implementation of current, evidence-based, treatment recommendations to optimize asthma management in this region.

**Trial registration:**

NCT03857178 (ClinicalTrials.gov).

**Supplementary Information:**

The online version contains supplementary material available at 10.1186/s40733-022-00085-5.

## Background

Asthma is one of the most common chronic respiratory diseases, estimated to affect 339 million people globally [1] and expected to rise to 400 million by 2025 [2, 3]. Although asthma research has received considerable attention worldwide, limited information on asthma management practices is available within the Gulf Cooperation Council (GCC) countries. The SNAPSHOT program, a cross-sectional, epidemiological study conducted between July 2014 and February 2016, offered insights on the prevalence of asthma within the Middle East, including Gulf countries [4]. At that time, the reported prevalence was 7.6% in Kuwait, Saudi Arabia, and the United Arab Emirates (UAE). A 2009 study estimated asthma prevalence among adults in Oman to be 7.3% [5]. Although the prevalence of asthma in the Middle East is lower than that in Europe and North America [4], uncontrolled disease continues to impose a substantial clinical and socioeconomic burden on patients, caregivers, and healthcare systems in Gulf nations [1, 6]. Moreover, between 2014 and 2015, the Epidemiological Study on the Management of Asthma in Asthmatic Middle East Adult Population (ESMAA) reported that approximately 60% of patients in both the UAE and Kuwait experienced uncontrolled or partly controlled asthma [7]. In the SNAPSHOT study, 38.2% of patients in Kuwait, Saudi Arabia, and UAE had uncontrolled asthma [6]. In addition, the Asthma Insights and Reality in the Gulf and the Near East (AIRGNE) study demonstrated that from January 2007 to March 2008, asthma control fell far below the goals of international guidelines, with 54% of patients with asthma in Oman described as poorly controlled or not well controlled [8]. Consequently, suboptimal disease control is likely to impose significant economic burdens on healthcare resource utilization in the Gulf region [9–12].

The AIRGNE-Oman study reported a frequency of hospitalization and emergency room (ER) visits in the preceding 12 months of 30% and 58%, respectively [8]. The 2009 total, annual, direct cost of asthma treatment in Oman was estimated to be more than Omani rial 61,500,294 (approximately 160 million United States dollars [USD]) [5]. Similarly, between 2009 and 2010, the total, annual, direct cost of asthma treatment in Kuwait was estimated to be more than 58 million Kuwaiti dinar (USD 207 million), of which 72% was allocated to inpatient and ER services [9].

Historically, short-acting β_2_-agonists (SABAs) have been prescribed for rapid symptomatic relief, despite their inherent lack of anti-inflammatory effects [13, 14]. Many patients with asthma residing in the Gulf region continue to rely on SABAs potentially at the expense of inhaled corticosteroids (ICS) [15]. This is concerning because SABA overuse, typically defined as the prescription or collection of ≥ 3 canisters per year [16], is associated with an increased risk of asthma exacerbations, hospitalizations, and mortality [17–19]. According to the 2010 Asthma Insights and Reality (AIR) survey conducted in the UAE, 67% of patients used SABAs, with only 5.5% using ICS in the preceding 12 months [15]. Another study reported that between 2014 and 2015, only 43.8% and 19.5% of patients in Kuwait and the UAE, respectively, used ICS/long-acting β_2_-agonist (ICS/LABA) fixed-dose combinations as their primary asthma treatment. These observations may explain, at least in part, the deficiencies in disease control, which persist in several countries in this region [7].

Since its landmark update in 2019, the Global Initiative for Asthma (GINA) no longer recommends as-needed SABAs without concomitant ICS. Rather, the revised strategy recommends low-dose ICS/formoterol as the preferred reliever for all patients with mild asthma and for patients with moderate-to-severe asthma who are prescribed ICS/formoterol maintenance and reliever therapy [20]. To effect change in clinical practice, these evidence-based treatment recommendations must be adopted at both national and local levels. However, the National Asthma Management Guidelines in Oman have not been updated since 2009 [21]. Moreover, neither Kuwait nor the UAE have established national or local asthma treatment guidelines. Data on prescription trends in asthma medications, especially the prevalence of SABA use and its implications, may provide clinicians with greater clarity on the extent of SABA overuse and encourage alignment of community practices with the latest evidence-based treatment recommendations [20].

Despite its associated disease burden, research on the management of asthma, including prescription patterns, and the effectiveness of asthma medications is limited within the GCC countries (including Bahrain, Kuwait, Oman, Qatar, Saudi Arabia, and the UAE) [22]. As part of the SABA use IN Asthma (SABINA) global studies [23], the international SABINA III study was initiated to understand SABA prescription volumes and associated clinical outcomes across 23 countries in the Asia Pacific, Africa, Latin America, the Middle East and in Russia [24]. Overall, findings from SABINA III in 8,351 patients demonstrated that SABA over-prescription, defined as ≥ 3 canisters in the course of 12 months, was common, occurring in 38% of patients, and was associated with increased incidence rates of severe asthma exacerbations and odds of inadequate disease control [24]. Here, we report the results from the Gulf cluster of SABINA III, comprising Kuwait, Oman, and the UAE, to provide real-world evidence on asthma management practices in this region.

## Methods

### Study design

SABINA III was a multicountry, multicenter, cross-sectional study conducted in 24 countries, which has been described previously [24]. Here, we report results from the Gulf cluster of Kuwait, Oman, and the UAE, with patients recruited in the 8 months between May and December 2019. The study sites are detailed in Supplementary Table 1. Consecutive patients attending health clinics were enrolled in the study. A clinical investigator at each site selected participants when they visited their doctor and met all eligibility criteria. No standardized method for this process was specified other than fulfilling the inclusion criteria. The primary objective of the study was to describe SABA prescription trends in the asthma patient population based on aggregated data collected from these three Gulf countries.

### Study population

Eligible patients were aged ≥ 12 years with a documented diagnosis of asthma, ≥ 3 consultations with a healthcare provider (HCP), and medical records containing data for ≥ 12 months before the study visit. Patients with a diagnosis of other chronic respiratory diseases, such as chronic obstructive pulmonary disease, or with a diagnosis of an acute or chronic condition that, in the opinion of the investigator, would limit the ability of the patient to participate in the study were excluded.

### Study variables

Each patient was classified by their SABA prescription volume during the 12 months preceding the study visit. SABA prescriptions were categorized as 0, 1–2, 3–5, 6–9, 10–12, and ≥ 13 canisters, and a prescription of ≥ 3 SABA canisters per year was defined as over-prescription [17, 18, 25]. Canisters of ICS prescribed in the prior 12 months also were recorded and categorized by their prescribed, average, daily dose of low, medium, or high based on GINA 2017 recommendations [13]. Patients were stratified by treatments prescribed in the 12 months prior to a single, prospective, study visit.

Secondary variables included practice type, i.e., either primary care or respiratory specialist; investigator-classified disease severity guided by GINA 2017 treatment steps, with steps 1–2 considered mild asthma and steps 3–5 considered moderate-to-severe asthma [13]; sociodemographic characteristics; duration of disease; and asthma treatments in the 12 months prior to the study visit, such as SABA monotherapy, SABA in addition to maintenance therapy, ICS/LABA fixed-dose combinations, oral corticosteroid (OCS) burst treatment, defined as a short course of intravenous corticosteroids or OCS administered for 3–10 days or a single dose of an intramuscular corticosteroid to treat an asthma exacerbation; antibiotics prescribed for asthma; and nonprescription SABA (over-the-counter [OTC]) purchases. Other variables included medication reimbursement status, number of comorbidities, and smoking status. Physicians entered data on prescriptions for asthma medication in the eCRF based on patient medical records.

### Outcomes

Asthma-related health outcomes included symptom control, assessed during the study visit, and the number of severe asthma exacerbations in the previous 12 months, which was collected based on data from patient medical records. Asthma symptom control was evaluated using the GINA 2017 assessment of asthma control and categorized as well controlled, partly controlled, or uncontrolled [13]. Severe exacerbation events were defined as a worsening of asthma symptoms that necessitated hospitalization, an ER visit, administration of an intravenous corticosteroid or OCS for ≥ 3 days, or single-dose administration of an intramuscular corticosteroid based on the American Thoracic Society/European Respiratory Society recommendations [26].

### Statistical analysis

Patient-level analyses were presented as descriptive statistics. Continuous variables were summarized as the number of non-missing values, mean (standard deviation [SD]), and median (range), and categorical variables were summarized as frequency counts and percentages.

## Results

### Patient disposition

**Fig. 1:**
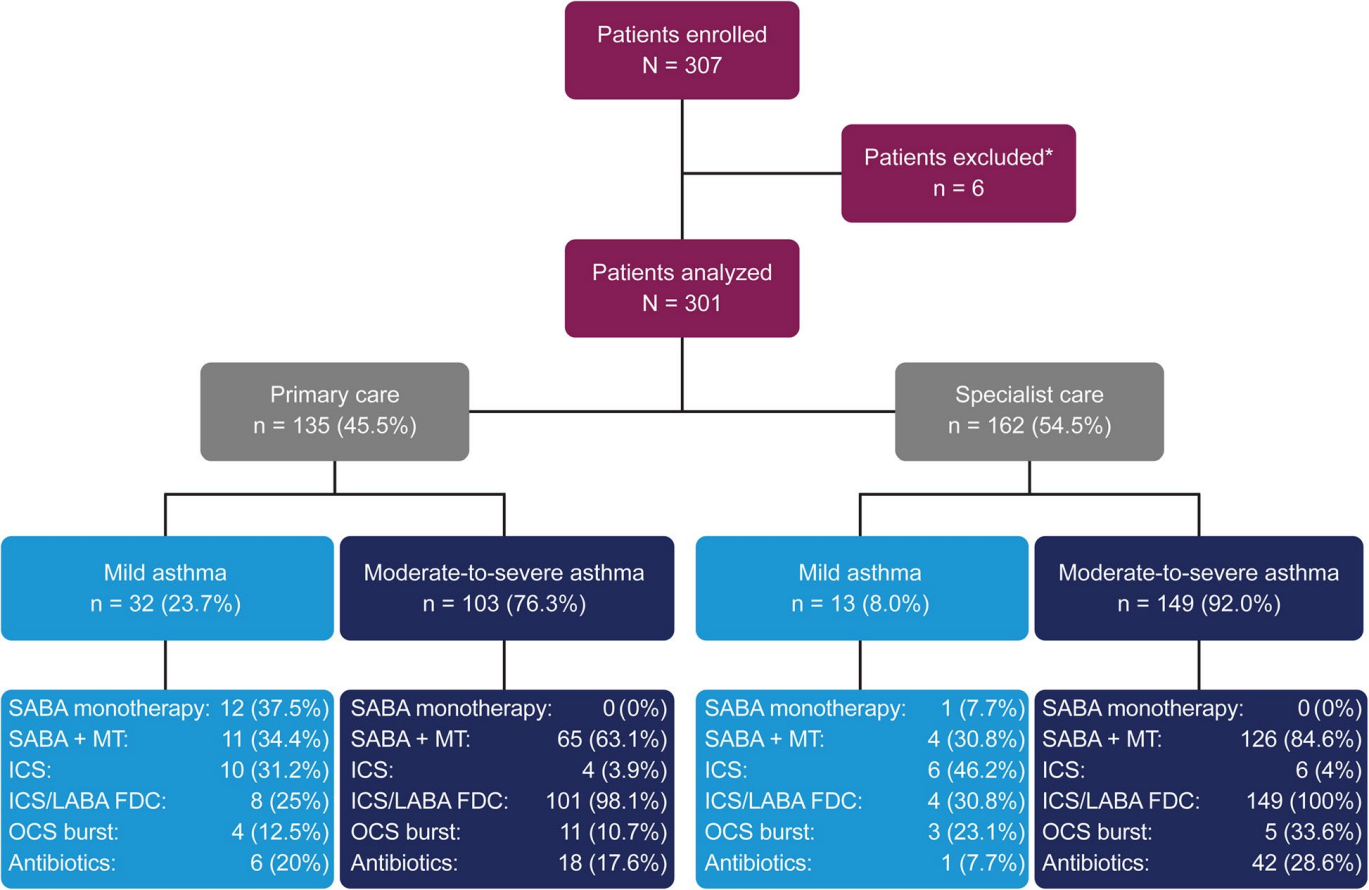
Patient disposition and study population by investigator-classified asthma severity in SABINA III Gulf cluster cohort. ^*^Patients with a history of asthma for < 12 months. Note: Prescriber type was not recorded for two patients each in mild asthma and moderate-to-severe asthma group. Patients could have been prescribed multiple treatments in the 12 months prior to the study visit. Abbreviations: FDC, fixed-dose combination; ICS, inhaled corticosteroids; LABA, long-acting β_2_-agonist; MT, maintenance therapy; OCS, oral corticosteroids; SABA, short-acting β_2_-agonist; SABINA, SABA use IN Asthma

Of the 307 patients enrolled in the study, six were excluded because they had an asthma duration of less than 12 months; therefore, a total of 301 patients were included in the analysis (Fig. 1). Most patients were recruited from Kuwait (*n* = 136), followed by the UAE (*n* = 122) and Oman (*n* = 43). A slightly higher proportion of patients were treated by respiratory specialists than by primary care physicians (54.5% and 45.5%, respectively).

### Patient and disease characteristics

Overall, the mean (SD) age of this study cohort was 43.9 (15.3) years, with most patients aged 18–54 years (68.1%) (Table 1). Most patients were female (61.8%), overweight or obese (81.4%), had a mean (SD) body mass index (BMI) of 30.2 (6.2) kg/m^2^, and had never smoked (85.7%). Nearly one-quarter of patients had received a high school education, while more than one-third had obtained a university and/or post-graduate education. In addition, most patients (95.7%) received full healthcare reimbursement, an observation that was consistent across practice types.

**Table 1:** Demographics and baseline clinical characteristics of the SABINA III Gulf cluster cohort

	**All** **(*N* = 301)**	**Primary care physicians (*n* = 135)**			**Respiratory specialists (*n* = 162)**		
		**Mild asthma** **(*n* = 32)**	**Moderate-to-severe asthma (*n* = 103)**	**All (*n* = 135)**	**Mild asthma** **(*n* = 13)**	**Moderate-to-severe asthma** **(*n* = 149)**	**All** **(*n* = 162)**
**Age, years**							
Mean (SD)	43.9 (15.3)	39.0 (14.9)	46.8 (15.7)	45.0 (15.8)	39.5 (7.9)	43.6 (15.3)	43.2 (14.9)
Median (min, max)	43.0 (12.0, 82.0)	40.5 (14.0, 71.0)	45.0 (12.0, 82.0)	45.0 (12.0, 82.0)	40.0 (23.0, 56.0)	44.0 (13.0, 74.0)	42.0 (13.0, 74.0)
**Age group, years, n (%)**							
12–17	18 (6.0)	3 (9.4)	5 (4.9)	8 (5.9)	0 (0)	10 (6.7)	10 (6.2)
18–54	205 (68.1)	25 (78.1)	68 (66)	93 (68.9)	12 (92.3)	96 (64.4)	108 (66.7)
≥ 55	78 (25.9)	4 (12.5)	30 (29.1)	34 (25.2)	1 (7.7)	43 (28.9)	44 (27.2)
**Sex, n (%)**							
Female	186 (61.8)	25 (78.1)	64 (62.1)	89 (65.9)	10 (76.9)	86 (57.7)	96 (59.3)
**BMI, kg/m**^**2**^							
Mean (SD)	30.2 (6.2)	31.5 (7.6)	30.2 (6.4)	30.5 (6.7)	30.1 (7.7)	29.8 (5.6)	29.9 (5.7)
Median (min, max)	29.4 (18.2, 53.9)	29.9 (21.2, 53.9)	29.1 (19.0, 52.3)	29.4 (19.0, 53.9)	29.9 (20.7, 47.1)	29.4 (18.2, 48.1)	29.4 (18.2, 48.1)
**BMI group, kg/m**^**2**^**, n (%)**							
< 18.5	2 (0.7)	0 (0)	0 (0)	0 (0)	0 (0)	2 (1.3)	2 (1.2)
≥ 18.5–24.9	54 (17.9)	6 (18.8)	22 (21.4)	28 (20.7)	3 (23.1)	23 (15.4)	26 (16)
≥ 25–29.9	107 (35.5)	10 (31.2)	35 (34)	45 (33.3)	4 (30.8)	56 (37.6)	60 (37)
≥ 30	138 (45.8)	16 (50)	46 (44.7)	62 (45.9)	6 (46.2)	68 (45.6)	74 (45.7)
**Education level, n (%)**							
Primary or secondary school	85 (28.2)	8 (25)	28 (27.2)	36 (26.7)	0 (0)	48 (32.2)	48 (29.6)
High school	73 (24.3)	4 (12.5)	30 (29.1)	34 (25.2)	6 (46.2)	33 (22.1)	39 (24.1)
University and/or post-graduate education	107 (35.5)	12 (37.5)	23 (22.3)	35 (25.9)	7 (53.8)	62 (41.6)	69 (42.6)
Unknown	36 (12)	8 (25)	22 (21.4)	30 (22.2)	0 (0)	6 (4)	6 (3.7)
**Healthcare insurance/medication funding, n (%)**							
Not reimbursed	0 (0)	0 (0)	0 (0)	0 (0)	0 (0)	0 (0)	0 (0)
Partially reimbursed	12 (4)	5 (15.6)	3 (2.9)	8 (5.9)	1 (7.7)	3 (2)	4 (2.5)
Fully reimbursed	287 (95.7)	27 (84.4)	99 (96.1)	126 (93.3)	12 (92.3)	145 (98)	157 (97.5)
Unknown	1 (0.3)	0 (0)	1 (1)	1 (0.7)	0 (0)	0 (0)	0 (0)
Missing values, n	1	0	0	0	0	1	1
**Smoking status history, n (%)**							
Active smoker	28 (9.3)	3 (9.4)	11 (10.7)	14 (10.4)	0 (0)	14 (9.4)	14 (8.6)
Former smoker	15 (5)	0 (0)	4 (3.9)	4 (3)	0 (0)	11 (7.4)	11 (6.8)
Never smoker	258 (85.7)	29 (90.6)	88 (85.4)	117 (86.7)	13 (100)	124 (83.2)	137 (84.6)

Prescriber type was not recorded for two patients each in mild asthma and moderate-to-severe asthma group

*Abbreviations: BMI* body mass index, *max* maximum, *min* minimum, *SABINA* SABA use IN Asthma, *SD* standard deviation

Most patients (84.4%) had investigator-classified moderate-to-severe asthma (GINA steps 3–5) and 15.6% had mild asthma (GINA steps 1–2), with a mean (SD) disease duration of 14.8 (10.8) years. The highest proportion of patients were at GINA treatment step 3 (41.5%) (Table 2). Overall, 41.2% of patients had no comorbidities, and 58.8% had ≥ 1 comorbidity. Patients reported a mean (SD) of 0.9 (1.6) severe exacerbations, with 41.9% experiencing ≥ 1 severe asthma exacerbation in the 12 months preceding the study visit (Table 2). Compared with patients treated by primary care physicians, a higher percentage of patients treated by respiratory specialists experienced ≥ 1 severe asthma exacerbation in the 12 months prior to their study visit (48.8% vs 34.8%). Asthma symptom control was assessed as well controlled in 48.8% of patients, partly controlled in 25.9% of patients, and uncontrolled in 25.2% of patients (Table 2). Compared with patients treated by primary care physicians, a higher proportion of those under respiratory specialist care had well-controlled asthma (54.3% vs 43.0%).

**Table 2:** Asthma characteristics of the SABINA III Gulf cluster cohort by investigator-classified asthma severity and practice type

	**All** **(*N* = 301)**	**Primary care physicians (*n* = 135)**			**Respiratory specialists (*n* = 162)**		
		**Mild asthma (*n* = 32)**	**Moderate-to-severe asthma (*n* = 103)**	**All** **(*n* = 135)**	**Mild asthma (*n* = 13)**	**Moderate-to-severe asthma (*n* = 149)**	**All** **(*n* = 162)**
**Asthma duration, years**							
Mean (SD)	14.8 (10.8)	15.2 (11.4)	14.4 (9.5)	14.6 (10.0)	10.2 (6.6)	15.2 (11.8)	14.8 (11.6)
Median (min, max)	12.0 (1.0, 50.0)	11.0 (1.0, 40.0)	13.0 (1.0, 39.0)	13.0 (1.0, 40.0)	7.0 (2.0, 21.0)	11.0 (1.0, 50.0)	11.0 (1.0, 50.0)
**Number of severe asthma exacerbations in the past 12 months**							
Mean (SD)	0.9 (1.6)	0.4 (1.0)	0.9 (1.5)	0.8 (1.4)	0.6 (1.0)	1.1 (1.8)	1.1 (1.8)
**Number of severe asthma exacerbations in the past 12 months by groups, n (%)**							
0	175 (58.1)	25 (78.1)	63 (61.2)	88 (65.2)	9 (69.2)	74 (49.7)	83 (51.2)
1	58 (19.3)	3 (9.4)	19 (18.4)	22 (16.3)	1 (7.7)	35 (23.5)	36 (22.2)
2	31 (10.3)	2 (6.2)	6 (5.8)	8 (5.9)	2 (15.4)	21 (14.1)	23 (14.2)
3	17 (5.6)	1 (3.1)	8 (7.8)	9 (6.7)	1 (7.7)	7 (4.7)	8 (4.9)
> 3	20 (6.6)	1 (3.1)	7 (6.8)	8 (5.9)	0 (0)	12 (8.1)	12 (7.4)
**Level of asthma symptom control, n (%)**							
Well controlled	147 (48.8)	16 (50)	42 (40.8)	58 (43)	11 (84.6)	77 (51.7)	88 (54.3)
Partly controlled	78 (25.9)	10 (31.2)	30 (29.1)	40 (29.6)	0 (0)	37 (24.8)	37 (22.8)
Uncontrolled	76 (25.2)	6 (18.8)	31 (30.1)	37 (27.4)	2 (15.4)	35 (23.5)	37 (22.8)
**GINA treatment step, n (%)**^**a**^							
Step 1	19 (6.3)	15 (46.9)	0 (0)	15 (11.1)	3 (23.1)	0 (0)	3 (1.9)
Step 2	28 (9.3)	17 (53.1)	0 (0)	17 (12.6)	10 (76.9)	0 (0)	10 (6.2)
Step 3	125 (41.5)	0 (0)	56 (54.4)	56 (41.5)	0 (0)	69 (46.3)	69 (42.6)
Step 4	78 (25.9)	0 (0)	36 (35)	36 (26.7)	0 (0)	40 (26.8)	40 (24.7)
Step 5	51 (16.9)	0 (0)	11 (10.7)	11 (8.1)	0 (0)	40 (26.8)	40 (24.7)
**Comorbidities, n (%)**							
None	124 (41.2)	11 (34.4)	42 (40.8)	53 (39.3)	5 (38.5)	63 (42.3)	68 (42)
1–2	137 (45.5)	18 (56.2)	45 (43.7)	63 (46.7)	8 (61.5)	65 (43.6)	73 (45.1)
3–4	37 (12.3)	3 (9.4)	15 (14.6)	18 (13.3)	0 (0)	19 (12.8)	19 (11.7)
≥ 5	3 (1)	0 (0)	1 (1)	1 (0.7)	0 (0)	2 (1.3)	2 (1.2)

^a^Based on 2017 GINA recommendations

Prescriber type was not recorded for two patients each in mild asthma and moderate-to-severe asthma group

*Abbreviations: GINA* Global Initiative for Asthma, *max* maximum, *min* minimum, *SABINA* SABA use IN Asthma, *SD* standard deviation

### Asthma treatments in the past 12 months

**Fig. 2:**
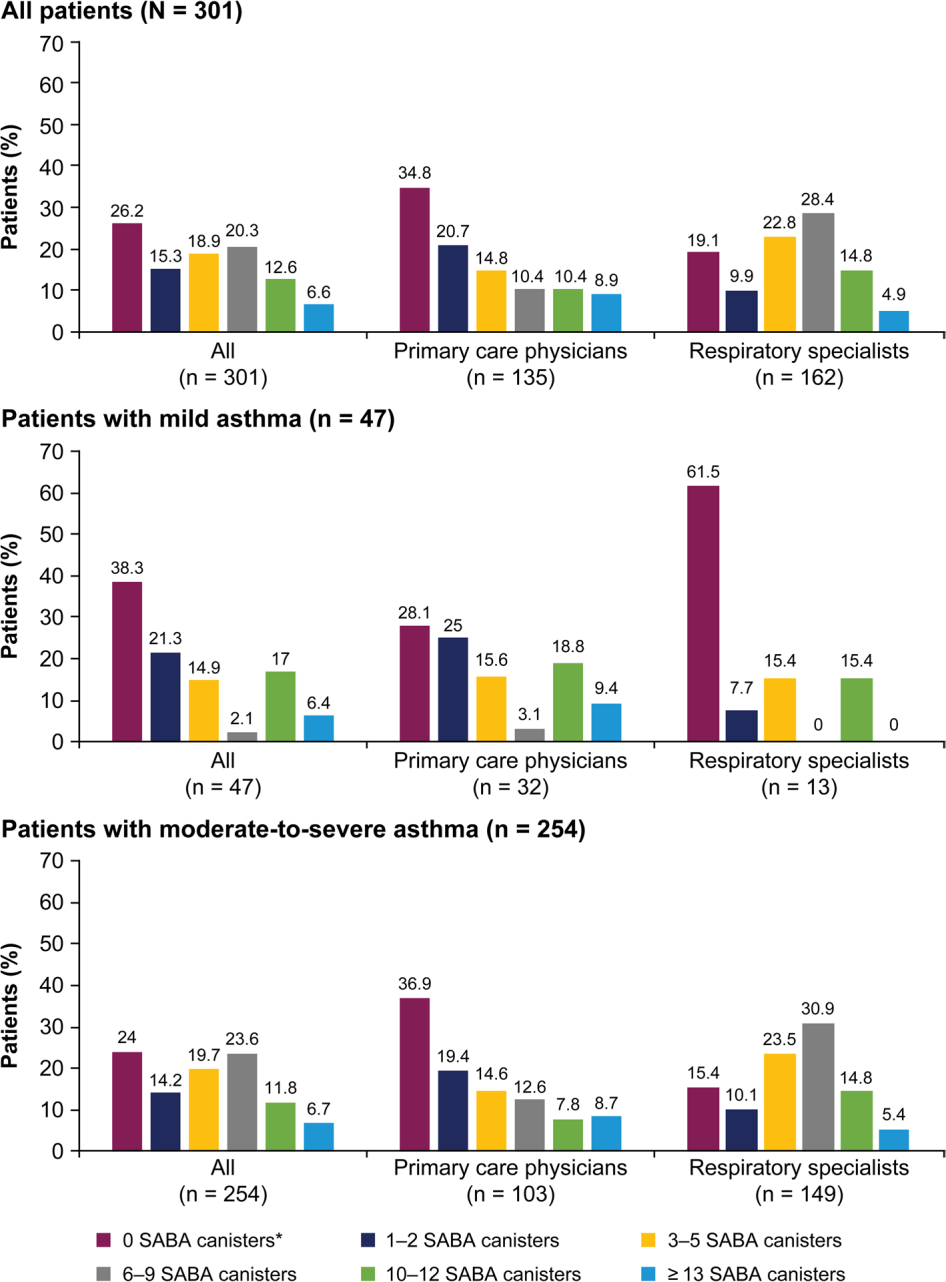
SABA prescriptions stratified by asthma severity in the SABINA III Gulf cluster cohort. *Patients without SABA prescriptions did not report the type of reliever they were using. Note: Prescriber type was not recorded for two patients each in the mild asthma and moderate-to-severe asthma group. Abbreviations: SABA, short-acting β_2_-agonist; SABINA, SABA use in Asthma

Overall, 58.5% of patients were prescribed ≥ 3 SABA canisters and 19.3% of patients were prescribed ≥ 10 SABA canisters in the 12 months before their study visit. Approximately one-fourth of all patients (26.2%) were prescribed 0 SABA canisters (Fig. 2). A higher percentage of patients treated by respiratory specialists were prescribed ≥ 3 SABA canisters in the preceding 12 months compared with patients treated by primary care physicians (71.0% vs 44.4%). Moreover, a higher proportion of patients classified with moderate-to-severe asthma were prescribed ≥ 3 SABA canisters in the 12 months prior to their study visit than patients with mild disease (61.8% vs 40.4%).

#### SABA monotherapy

Of 301 enrolled patients, only 14 patients (4.7%) were prescribed SABA monotherapy, with a mean (SD) of 8.9 (12.9) canisters in the 12 months preceding their study visit (Table 3). Among patients under primary care, 8.9% (*n* = 12), and all classified with mild asthma, were prescribed SABA monotherapy, with a mean (SD) of 10.2 (13.5) canisters in the 12 months before their study visit. Only one patient (0.6%) treated by a respiratory specialist and classified with mild asthma was prescribed a single canister of SABA monotherapy.

**Table 3:** SABA Prescriptions in the past 12 months for the SABINA III Gulf cluster cohort

	**All** **(*N* = 301**)	**Primary care physicians (*n* = 135)**			**Respiratory specialists (*n* = 162)**		
		**Mild asthma (*n* = 32)**	**Moderate-to-severe asthma** **(*n* = 103)**	**All** **(*n* = 135)**	**Mild asthma (*n* = 13)**	**Moderate-to-severe asthma** **(*n* = 149)**	**All** **(*n* = 162)**
**Prescription of SABA monotherapy, n (%)**							
No	287 (95.3)	20 (62.5)	103 (100)	123 (91.1)	12 (92.3)	149 (100)	161 (99.4)
Yes	14 (4.7)	12 (37.5)	0 (0)	12 (8.9)	1 (7.7)	0 (0)	1 (0.6)
***SABA canisters or inhalers prescribed in the past 12 months***							
Mean (SD)	8.9 (12.9)	10.2 (13.5)	NA	10.2 (13.5)	1.0 (NA)	NA	1.0 (NA)
Median (min, max)	2.5 (1.0, 42.0)	3.0 (1.0, 42.0)	NA	3.0 (1.0, 42.0)	1.0 (1.0, 1.0)	NA	1.0 (1.0, 1.0)
***SABA canisters or inhalers prescribed in the past 12 months by groups, n (%)***							
0–2	7 (50)	5 (41.7)	NA	5 (41.7)	1 (100)	NA	1 (100)
3–5	3 (21.4)	3 (25)	NA	3 (25)	0 (0)	NA	0 (0)
6–9	0 (0)	0 (0)	NA	0 (0)	0 (0)	NA	0 (0)
10–12	1 (7.1)	1 (8.3)	NA	1 (8.3)	0 (0)	NA	0 (0)
≥ 13	3 (21.4)	3 (25)	NA	3 (25)	0 (0)	NA	0 (0)
**Prescription of SABA in addition to maintenance therapy/SABA add-on therapy, n (%)**							
No	93 (30.9)	21 (65.6)	38 (36.9)	59 (43.7)	9 (69.2)	23 (15.4)	32 (19.8)
Yes	208 (69.1)	11 (34.4)	65 (63.1)	76 (56.3)	4 (30.8)	126 (84.6)	130 (80.2)
***SABA canisters or inhalers prescribed in the past 12 months***							
Mean (SD)	8.0 (10.9)	7.6 (4.6)	10.6 (18.5)	10.2 (17.2)	7.8 (4.3)	6.8 (4.0)	6.8 (4.0)
Median (min, max)	6.0 (1.0, 110.0)	9.0 (2.0, 12.0)	5.0 (1.0, 110.0)	5.0 (1.0, 110.0)	7.5 (4.0, 12.0)	6.0 (1.0, 24.0)	6.0 (1.0, 24.0)
***SABA canisters or inhalers prescribed in the past 12 months by groups, n (%)***							
0–2	39 (18.8)	3 (27.3)	20 (30.8)	23 (30.3)	0 (0)	15 (11.9)	15 (11.5)
3–5	54 (26)	2 (18.2)	15 (23.1)	17 (22.4)	2 (50)	35 (27.8)	37 (28.5)
6–9	61 (29.3)	1 (9.1)	13 (20)	14 (18.4)	0 (0)	46 (36.5)	46 (35.4)
10–12	37 (17.8)	5 (45.5)	8 (12.3)	13 (17.1)	2 (50)	22 (17.5)	24 (18.5)
≥ 13	17 (8.2)	0 (0)	9 (13.8)	9 (11.8)	0 (0)	8 (6.3)	8 (6.2)

Prescriber type was not recorded for two patients each in mild asthma and moderate-to-severe asthma group

*Abbreviations: max* maximum, *min* minimum, *NA* not available, *SABA* short-acting β_2_-agonist, *SABINA* SABA use IN Asthma, *SD* standard deviation

#### SABA in addition to maintenance therapy

Overall, 69.1% of patients (*n* = 208) were prescribed SABA in addition to maintenance therapy, with a mean (SD) of 8.0 (10.9) canisters in the 12 months before the study visit (Table 3). Among these patients, 81.3% were prescribed ≥ 3 SABA canisters and 26.0% were prescribed ≥ 10 SABA canisters in the previous 12 months. A higher proportion of patients treated by respiratory specialists were prescribed ≥ 3 SABA canisters compared with patients treated by primary care physicians (88.5% vs 69.7%), whereas a comparable proportion of patients treated by respiratory specialists and primary care physicians were prescribed ≥ 10 SABA canisters in the 12 months preceding their study visit (24.6% vs 28.9%).

#### SABA OTC without a prescription

Overall, 13.3% (*n* = 40) of patients purchased SABA OTC in the 12 months preceding the study visit, with 52.5% (*n* = 21) purchasing ≥ 3 SABA canisters (Table 4). SABA OTC purchase was observed to be greater among patients treated by respiratory specialists than among those treated by primary care physicians (19.1% vs 5.9%); all patients who obtained SABA OTC while under respiratory specialist care had been classified with moderate-to-severe asthma. In addition, a higher proportion of patients receiving care from a respiratory specialist purchased ≥ 3 SABA canisters in the prior 12 months compared with patients receiving treatment from primary care physicians (54.8% vs 37.5%). Further, among those patients who purchased SABA OTC (*n* = 40), the majority (92.5%, *n* = 37) also received SABA prescriptions. More than half of these patients (51.4%) received ≥ 3 SABA prescriptions and 13.5% received ≥ 10 SABA prescriptions.

**Table 4:** SABA OTC purchase in the past 12 months in the SABINA III Gulf cluster cohort

	**All** **(*N* = 301**)	**Primary care physicians (*n* = 135)**			**Respiratory specialists (*n* = 162)**		
		**Mild asthma (*n* = 32)**	**Moderate-to-severe asthma** **(*n* = 103)**	**All** **(*n* = 135)**	**Mild asthma** **(*n* = 13)**	**Moderate-to-severe asthma** **(*n* = 149)**	**All** **(*n* = 162)**
**Patients who purchased SABA without a prescription in the past 12 months, n (%)**							
No	244 (81.1)	27 (84.4)	83 (80.6)	110 (81.5)	13 (100)	118 (79.2)	131 (80.9)
Yes	40 (13.3)	1 (3.1)	7 (6.8)	8 (5.9)	0 (0)	31 (20.8)	31 (19.1)
Unknown	17 (5.6)	4 (12.5)	13 (12.6)	17 (12.6)	0 (0)	0 (0)	0 (0)
***Number of SABA canisters or inhalers purchased without prescriptions in the past 12 months, n (%)***							
1–2	15 (37.5)	1 (100)	3 (42.9)	4 (50)	NA	11 (35.5)	11 (35.5)
3–5	13 (32.5)	0 (0)	2 (28.6)	2 (25)	NA	10 (32.3)	10 (32.3)
6–9	2 (5)	0 (0)	0 (0)	0 (0)	NA	2 (6.5)	2 (6.5)
10–12	4 (10)	0 (0)	0 (0)	0 (0)	NA	4 (12.9)	4 (12.9)
≥ 13	2 (5)	0 (0)	1 (14.3)	1 (12.5)	NA	1 (3.2)	1 (3.2)
Not applicable^a^	4 (10)	0 (0)	1 (14.3)	1 (12.5)	NA	3 (9.7)	3 (9.7)

^a^“﻿Not applicable” could be selected in the eCRF when patients purchased non-canister forms of SABA (e.g., oral, or nebulized SABA) without a prescription

Prescriber type was not recorded for 2 patients each in mild asthma and moderate-to-severe asthma group

*Abbreviations: NA* not available, *OTC* over the counter, *SABA* short-acting β_2_-agonist, *SABINA* SABA use IN Asthma, *eCRF* electronic case report form

#### Prescriptions of other asthma medications

Few patients [9% (*n* = 27)] were prescribed ICS monotherapy, with a median (range) of 12.0 (1.0, 110.0) canisters in the 12 months before the study visit (Table 5). Most patients were prescribed medium-dose ICS (48.1%), while 44.4% (*n* = 12) and 7.4% (*n* = 2) of patients were prescribed low-dose and high-dose ICS, respectively.

**Table 5:** Prescription of other medications in the past 12 months in the SABINA III Gulf cluster cohort

	**All** **(*N* = 301**)	**Primary care physicians (*n* = 135)**			**Respiratory specialists (*n* = 162)**		
		**Mild asthma (*n* = 32)**	**Moderate-to-severe asthma** **(*n* = 103)**	**All** **(*n* = 135)**	**Mild asthma (*n* = 13)**	**Moderate-to-severe asthma** **(*n* = 149)**	**All** **(*n* = 162)**
**Prescription of ICS, n (%)**							
No	274 (91)	22 (68.8)	99 (96.1)	121 (89.6)	7 (53.8)	143 (96)	150 (92.6)
Yes	27 (9)	10 (31.2)	4 (3.9)	14 (10.4)	6 (46.2)	6 (4)	12 (7.4)
***ICS canisters or inhalers prescribed in the past 12 months***							
Mean (SD)	16.8 (20.9)	26.7 (32.5)	8.2 (7.5)	21.4 (28.6)	11.8 (2.9)	11.7 (0.8)	11.8 (2.0)
Median (min, max)	12.0 (1.0, 110.0)	12.0 (2.0, 110.0)	7.5 (1.0, 17.0)	12.0 (1.0, 110.0)	12.0 (7.0, 16.0)	12.0 (10.0, 12.0)	12.0 (7.0, 16.0)
**Total daily ICS dose, n (%)**							
Low dose	12 (44.4)	6 (60)	2 (50)	8 (57.1)	2 (33.3)	1 (16.7)	3 (25)
Medium dose	13 (48.1)	3 (30)	2 (50)	5 (35.7)	4 (66.7)	4 (66.7)	8 (66.7)
High dose	2 (7.4)	1 (10)	0 (0)	1 (7.1)	0 (0)	1 (16.7)	1 (8.3)
**Prescription of ICS/LABA fixed-dose combination, n (%)**							
No	37 (12.3)	24 (75)	2 (1.9)	26 (19.3)	9 (69.2)	0 (0)	9 (5.6)
Yes	264 (87.7)	8 (25)	101 (98.1)	109 (80.7)	4 (30.8)	149 (100)	153 (94.4)
***Total daily ICS dose, n (%)***							
Low dose	99 (37.8)	6 (75)	42 (41.6)	48 (44)	0 (0)	51 (34.5)	51 (33.6)
Medium dose	114 (43.5)	2 (25)	48 (47.5)	50 (45.9)	4 (100)	59 (39.9)	63 (41.4)
High dose	49 (18.7)	0 (0)	11 (10.9)	11 (10.1)	0 (0)	38 (25.7)	38 (25)
Missing values, n	2	0	0	0	0	1	1
**Prescription of OCS burst treatment/short course, n (%)**							
No	233 (77.4)	28 (87.5)	92 (89.3)	120 (88.9)	10 (76.9)	99 (66.4)	109 (67.3)
Yes	68 (22.6)	4 (12.5)	11 (10.7)	15 (11.1)	3 (23.1)	50 (33.6)	53 (32.7)
***Total daily dose, mg/day***							
Mean (SD)	37.0 (30.4)	38.3 (18.9)	58.3 (71.7)	54.0 (63.9)	28.3 (12.6)	32.8 (7.4)	32.5 (7.6)
Median (min, max)	30.0 (4.0, 200.0)	30.0 (25.0, 60.0)	27.0 (4.0, 200.0)	28.5 (4.0, 200.0)	30.0 (15.0, 40.0)	30.0 (15.0, 40.0)	30.0 (15.0, 40.0)
***Number of days per prescription, n (%)***							
Mean (SD)	5.1 (2.0)	5.0 (0.0)	5.1 (2.9)	5.1 (2.5)	4.7 (0.6)	5.2 (1.9)	5.1 (1.9)
Median (min, max)	5.0 (1.0, 15.0)	5.0 (5.0, 5.0)	5.0 (1.0, 10.0)	5.0 (1.0,10.0)	5.0 (4.0, 5.0)	5.0 (1.0, 15.0)	5.0 (1.0, 15.0)
**Prescription of antibiotics for asthma, n (%)**							
No	229 (77.4)	24 (80)	84 (82.4)	108 (81.8)	12 (92.3)	105 (71.4)	117 (73.1)
Yes	67 (22.6)	6 (20)	18 (17.6)	24 (18.2)	1 (7.7)	42 (28.6)	43 (26.9)
Missing values, n	5	2	1	3	0	2	2

Prescriber type was not recorded for two patients each in mild asthma and moderate-to-severe asthma group

*Abbreviations: ICS* inhaled corticosteroids, *LABA* long-acting β_2_-agonist, *max* maximum, *min* minimum, *OCS* oral corticosteroids, *SABINA* SABA use IN Asthma, *SD* standard deviation

Of the entire cohort, 87.7% of patients (*n* = 264) were prescribed an ICS/LABA fixed-dose combination (Table 5). Of these patients, most were prescribed medium-dose ICS (43.5%), while 37.8% (*n* = 99) and 18.7% of patients (*n* = 49) were prescribed low-dose ICS and high-dose ICS, respectively.

During the 12 months preceding the study visit, an OCS burst was prescribed to 22.6% of all patients (Table 5). A higher proportion of patients treated by respiratory specialists were prescribed an OCS burst compared with those treated by primary care physicians (32.7% vs 11.1%).

In total, 22.6% of patients (*n* = 67) were prescribed antibiotics for asthma, most of whom had been classified with moderate-to-severe disease (Table 5). It was found that a higher percentage of respiratory specialists were more likely to prescribe antibiotics compared with primary care physicians (26.9% vs 18.2%).

#### Severe exacerbations stratified by asthma treatment

When patients were stratified by treatments prescribed in the 12 months prior to their study visit, most patients prescribed an OCS burst had experienced ≥ 1 severe exacerbation (79.4%), followed by those prescribed antibiotics (67.2%); SABA in addition to maintenance therapy (49.5%); ICS/LABA fixed-dose combination (43.6%); ICS (33.3%); and SABA monotherapy (21.4%). However, it should be noted that in this study, not all patients who received OCS burst therapy reported a relevant asthma exacerbation, suggesting the need for improved reporting of exacerbation events.

## Discussion

Findings from the Gulf cohort of the SABINA III study provide valuable, real-world evidence on asthma management practices in this region. Widespread SABA over-prescription was observed with 58.5% of patients prescribed ≥ 3 SABA canisters and 19.3% prescribed ≥ 10 canisters in the 12 months preceding the study visit, which punctuates the significant disease burden imposed by asthma on the patient population in this region. Notably, among patients prescribed SABA in addition to maintenance therapy, more than 80% were prescribed ≥ 3 SABA canisters and 26% were prescribed ≥ 10 canisters. This observation emphasizes the gap in awareness with respect to the implementation of evidence-based treatment and prevention strategies in certain parts of the Gulf region. A recent retrospective study of patients with asthma who attended a pulmonology or allergy clinic from May 2015 to December 2019 reported a 65.3% misclassification of disease severity as “﻿severe asthma”﻿ rather than mild or moderate asthma based on GINA recommendations [27]. This observed inaccuracy might be ascribed to a lack of awareness, understanding, and/or adoption of GINA treatment strategies by clinicians compounded by patient noncompliance with current therapeutic recommendations. Indeed, SABA over-prescription in the Gulf cluster was considerably higher than that reported in the SABINA III multicountry, cross-sectional study (58.5% vs 38.0%) [24], which underscores the urgent need for improved asthma management practices in the Gulf region. In parallel, asthma-related clinical outcomes were found to be suboptimal, with more than half of all patients having partly controlled or uncontrolled disease and 41.9% experiencing ≥ 1 severe asthma exacerbation in the 12 months prior to their study visit.

The patient profiles between the Gulf cluster and the SABINA III population were generally consistent [24], with a few notable exceptions. The mean BMI of patients was higher in the Gulf cohort than in the SABINA III population (30.2 kg/m^2^ vs 27.8 kg/m^2^), and a higher proportion of patients (81.4% vs 65.6%) were overweight or obese in this region. Moreover, the proportions of overweight and obese patients with asthma were also higher than those reported in the SNAPSHOT observational study conducted in five countries, including Kuwait and the UAE [6]. These findings corroborate those of other studies, which report obesity levels having attained epidemic proportions in Gulf countries [28–30]. The higher prevalence of obesity might also be explained in part by a 61.8% representation of female patients in this cohort, as older women with a high BMI represent a unique cluster of patients with asthma [31].

In contrast to the aggregated SABINA III data, in which more than 80% of patients were enrolled by respiratory specialists and thus may have constituted an overall “better case scenario,” [24] the distribution of patients receiving primary and respiratory specialist care (45.5% and 54.5%, respectively) was well balanced in the Gulf region cohort, providing a more equitable assessment of asthma management practices in this region. A similar distribution of patients with asthma treated by a pulmonologist was also observed in a recent subset analysis from the Asthma Insights and Management (AIM) survey in the UAE (61.0%) and Kuwait (48.0%) [32]. Interestingly, despite the relatively even distribution of patients among primary care physicians, internists, and respiratory specialists, including allergists (41.4% vs 51.4%), most patients in this cohort (84.4%) were classified with moderate-to-severe disease, which may reflect the high asthma morbidity in the region, or a misperception of severity based on questionnaire data, or incorrect entries on medical records [27, 32]. Notably, 13.3% of patients reported purchasing SABA OTC, of whom 52.5% purchased ≥ 3 SABA canisters in the prior 12 months. This indicates an additional avenue for SABA canister distribution but is of particular concern because an increased reliance on SABA OTC is associated with increased ER visits and low adherence to prescription medication, thus contributing to poor asthma control [33, 34]. In addition, most patients who purchased SABA OTC also had received SABA prescriptions, further accentuating SABA overuse in this region. The high rate of SABA OTC purchases might be explained by “medicine sharing” practices commonly observed in this region [35, 36]. An additional factor that may contribute to SABA overuse is the cultural norm of “doctor shopping,” defined as seeking healthcare from multiple facilities. This is a common practice in the Gulf countries wherein patients avail themselves of additional SABA prescriptions from multiple HCPs. The subsequent overlap of SABA prescriptions and OTC purchases is fostered by a lack of inter-healthcare facility communication. These findings substantiate the need to stimulate policy changes that regulate SABA purchases with and without prescriptions to ensure optimal management for all patients with asthma. Recently, Dubai introduced a health information exchange platform (NABIDH) that offers bidirectional communication between healthcare institutions to improve health-related outcomes [37].

While previous studies have not assessed the extent of SABA over-prescription in Kuwait, Oman, and the UAE, our findings are consistent with those that suggest a history of over-reliance on SABA in the Gulf region. In 2012, a 3-month observational study in outpatient respiratory disease clinics in Dubai, UAE, reported that more than 40% of patients with asthma were prescribed SABA, thus making it the most prescribed class of medication in that country [38]. Similarly in Oman, a cross-sectional study demonstrated that SABA inhalers are highly prescribed in patients with asthma. A total of 93% and 82% of patients with asthma were prescribed SABA inhalers in asthma clinics and general medicine clinics located within the same primary health care centers, respectively [39]. The AIRGNE study also indicated that 92% of Omanis with asthma rely on rapid relief medications, such as SABA [8]. Over-prescription of SABA clearly suggests suboptimal treatment of this disease in the Gulf region, even among patients under the care of a respiratory specialist. However, it should be noted that data for this study were collected prior to the 2019 updated GINA report, which no longer recommends treatment with SABAs without concomitant ICS [20]. Nonetheless, as nearly 6 of 10 patients were overprescribed SABA in the Gulf cluster, our findings imply ingrained physician and patient behavior that likely will require targeted educational and outreach initiatives supported by government policies to effect changes in asthma prescribing practices and improve clinical outcomes.

Most patients (87.7%) in this cohort were prescribed ICS/LABA fixed-dose combinations as maintenance therapy, which aligns with the classification of moderate-to-severe asthma in the majority of study participants (84.4%). Compared with previous studies in the GCC countries, we demonstrated a significant increase in the usage of ICS/LABA fixed-dose combinations in patients with asthma (76.8% in Kuwait and 73.1% in the UAE) [22]. Similarly, in the AIM study, 80.8% of survey respondents (*n* = 574) reported most recently using long-term maintenance therapy within the past four weeks [40]. Leukotriene receptor antagonists [67.7% (*n* = 433)], ICS/LABA combinations [47.3% (*n* = 303)], and budesonide inhalation suspension [10.5% (*n* = 67)] comprised the largest selection of anti-inflammatory, controller agents. On the other hand, the AIRGNE study revealed a nominal use of preventive ICS (5%) in patients with asthma in Oman, which was one of the lowest within the study [8]. Thus, while our data suggest that currently more patients are prescribed anti-inflammatory maintenance therapy in this region, the accompanying over-prescription of SABA remains a concern, particularly since SABA overuse is associated with poor asthma outcomes even after adjusting for ICS adherence [17].

Overall, 22.6% of patients were prescribed an OCS burst, most likely for management of a severe asthma exacerbation, as 79.4% of patients with OCS short burst prescriptions had ≥ 1 exacerbation in the 12 months before their study visit. Indeed, similar findings were reported in a 2018 study, which characterized patients with severe asthma in eight countries, including the UAE [41]. In that study, physicians reported that 25% of patients with uncontrolled asthma in the UAE were OCS users, approximately one-third of whom chronically used oral corticosteroids [41]. The relatively high percentage of patients prescribed OCS could be explained, in part, by the practice of prescribing OCS as a prophylactic medication to prevent future asthma exacerbations [42]. However, the recurrent use of OCS should be limited, as it is associated with serious adverse effects and a low maintenance therapy-to-total prescription ratio [43–45]. Moreover, existing evidence does not support the benefits associated with regular OCS use in patients with asthma outside the episodes of exacerbations [46]. A total of 22.6% of patients were prescribed antibiotics for asthma, with a higher rate of prescription by respiratory specialists. This result further illustrates the noncompliance with international, evidence-based asthma treatment strategies, as GINA does not recommend concomitant antibiotics unless there is strong evidence of a bacterial respiratory infection [47]. This finding is of particular interest in the UAE, as there is evidence of highly prevalent self-medication with antibiotics despite the enforcement of new legislation prohibiting the sale of antibiotics without a prescription [48].

While the proportion of patients with well-controlled asthma in our study (48.8%) was greater than that in a previous study (29.4%) in the region [7], there remains opportunity for practice improvement, especially given the high rate of asthma exacerbations. The level of asthma control is impacted by several factors, including adoption of currently applicable guidelines as well as education on disease- and treatment-related aspects of asthma care [49]. Indeed, a recent systematic review of 13 studies conducted in the Gulf countries identified asthma-related education on the disease state; asthma medications; correct inhaler technique; prevention and treatment of asthma symptoms; perception of the role of ICS; and attitudes toward ER visits for first-line asthma care as one of the most common determinants of asthma control [22]. These findings exemplify the critical role that health literacy and education play in improving asthma care [7, 8]. Suboptimal asthma treatment outcomes as observed in the Gulf cluster might be further illuminated by the SNAPSHOT study, which reported that 30% of patients with asthma were followed by a physician in the UAE, Kuwait, and Saudi Arabia. However, 86.9% of patients in the Gulf region sought unscheduled healthcare visits, and more importantly, 36.5% of patients failed to seek any clinical consultation [6]. Uncontrolled asthma in Kuwait has also been attributed to the preference of patients in the region to visit hospital-based physicians and ERs, as Kuwaitis receive universal healthcare coverage. However, sporadic and unscheduled hospital visits for new or worsening asthma symptoms do not afford physicians the opportunity to educate patients and develop action plans for individualized disease management [50]. Of note, despite more patients in specialist care reporting well-controlled asthma compared with those treated in primary care (54.3% vs 43.0%), a relatively greater proportion of specialist-care treated patients experienced ≥ 1 severe exacerbation in the prior year (48.8% vs 34.8%). This could be attributed to more patients being classified with moderate-to-severe asthma in specialist vs primary care (92.0% vs 76.3%) and the greater SABA over-prescription observed (71.0% vs 44.4%). Indeed, several studies have reported similar findings, with patients with severe asthma frequently experiencing poor symptom control [51, 52].

There are some limitations to this multicountry cohort study. Prescription data were used as a surrogate for actual medication use and as such provide no information on medication adherence, which potentially contributes to an under-estimation or over-estimation of SABA use. However, SABA over-prescriptions have been linked to a risk of asthma exacerbations and inadequate disease control [24], corroborating the validity of this concern and likely suggesting actual medication use. Because data transcription onto the electronic case report form relied on clinical assessment, findings may have been impacted by misinterpretation of instructions and recall bias for data obtained directly from the patients such as SABA OTC use and asthma control. Comparisons across asthma severities were not made because most recruited patients were classified with moderate-to-severe disease. As only three Gulf countries were included in this analysis, the results may not be generalizable across the entire region. Despite these limitations, to our knowledge, this study provides a comprehensive assessment of SABA prescription volumes, SABA OTC purchases, and asthma outcomes in the three countries of Kuwait, Oman, and the UAE.

Primary, real-world data on SABA over-prescription in patients with asthma distributed across primary and respiratory specialist care represents contemporary asthma management practices and prescribing habits in the region. The findings from this study are particularly relevant considering the paradigm shift in asthma treatment guidelines with respect to SABA monotherapy [53] and emphasize the need for clinicians and policymakers alike to institute targeted improvements in prescribing and dispensing practices that align with the latest evidence-based asthma treatment guidelines. This progress calls for corresponding educational initiatives aimed at patients, pharmacists, and physicians, which focus on the heterogeneity of this chronic respiratory disease, updated treatment guidelines that address the spectrum of asthma severity, adverse effects of SABA overuse, and the availability of alternative, anti-inflammatory reliever therapy with the principal goal of optimizing asthma treatment and its sustained control.

## Conclusions

Results from the SABINA III Gulf cluster cohort in Kuwait, Oman, and the UAE revealed that 58.5% of study participants were overprescribed SABA (≥ 3 canisters per year) in the 12 months preceding their study visit. SABA over-prescription occurred commonly, irrespective of prescriber type. The disease burden was high, with less than half of all patients achieving acceptable control, and 41.9% experiencing ≥ 1 severe asthma exacerbation in the preceding 12 months. As SABA over-prescriptions have been linked to suboptimal clinical outcomes [17, 24], these findings support the position that asthma represents a major public health concern in the Gulf cluster. Our findings serve as a call to action for all stakeholders invested in achieving optimal care and clinical outcomes for all patients with asthma throughout this region, including HCPs and policymakers, to collaborate in ensuring that clinical practice aligns with the latest evidence-based treatment recommendations to achieve these paramount public health objectives.

## Supplementary Information

**Additional file 1: Supplementary Table 1. **Study sites in SABINA Gulf study.

## Data Availability

Data underlying the findings described in this manuscript may be obtained in accordance with AstraZeneca’s data sharing policy described at https://astrazenecagrouptrials.pharmacm.com/ST/Submission/Disclosure.

## Abbreviations

AIM: Asthma Insights and Management
AIR: Asthma Insights and Reality
AIRGNE: Asthma Insights and Reality in the Gulf and the Near East
BMI: Body mass index
ER: Emergency room
ESMAA: Epidemiological Study on the Management of Asthma in an Asthmatic Middle East Adult Population
GCC: Gulf Cooperation Council
GINA: Global Initiative for Asthma
HCP: Healthcare provider
ICS: Inhaled corticosteroids
LABA: Long-acting β_2_-agonist
OCS: Oral corticosteroids
OTC: Over the counter
SABA: Short-acting β_2_-agonist
SABINA: SABA use IN Asthma
SD: Standard deviation
UAE: United Arab Emirates
USD: United States dollar

## References

[1] Global Asthma Network (GAN). The Global Asthma Report (2018). http://www.globalasthmareport.org. Accessed 22 Dec 2021.

[2] Masoli M, Fabian D, Holt S, Beasley R, Global Initiative for Asthma Program (2004). The global burden of asthma: executive summary of the GINA Dissemination Committee Report. Allergy..

[3] World Health Organization (WHO). Global surveillance, prevention and control of chronic respiratory diseases: a comprehensive approach (2007). https://www.who.int/gard/publications/GARD_Manual/en/. Accessed 22 Dec 2021.

[4] Tarraf H, Aydin O, Mungan D, Albader M, Mahboub B, Doble A (2018). Prevalence of asthma among the adult general population of five Middle Eastern countries: results of the SNAPSHOT program. BMC Pulm Med.

[5] Al-Busaidi NH, Habibullah Z, Soriano JB (2013). The asthma cost in oman. Sultan Qaboos Univ Med J.

[6] Mungan D, Aydin O, Mahboub B, Albader M, Tarraf H, Doble A (2018). Burden of disease associated with asthma among the adult general population of five Middle Eastern countries: Results of the SNAPSHOT program. Respir Med.

[7] Tarraf H, Al-Jahdali H, Al Qaseer AH, Gjurovic A, Haouichat H, Khassawneh B (2018). Asthma control in adults in the Middle East and North Africa: Results from the ESMAA study. Respir Med.

[8] Al-Busaidi N, Soriano JB (2011). Asthma control in Oman: National results within the Asthma Insights and Reality in the Gulf and the Near East (AIRGNE) Study. Sultan Qaboos Univ Med J.

[9] Khadadah M (2013). The cost of asthma in Kuwait. Med Princ Pract.

[10] Al-Busaidi N, Habibulla Z, Bhatnagar M, Al-Lawati N, Al-Mahrouqi Y (2015). The burden of asthma in Oman. Sultan Qaboos Univ Med J.

[11] Al Mazrouei K, Almannaei AI, Nur FM, Bachnak N, Alzaabi A (2021). direct and indirect costs of asthma burden in abu Dhabi: A Retrospective Analysis of Insurance Claims Data from 2015 to 2018. Clinicoecon Outcomes Res.

[12] Mahboub BH, Safarini B, AbdulAziz M, Mustafa G. Cost of asthma in Dubai, United Arab Emirates (UAE). J Pulmon Resp Med. 2013;3.

[13] Global Initiative for Asthma (GINA). Global Strategy for Asthma Management and Prevention (2017). https://ginasthma.org/gina-reports/. Accessed 22 Dec 2021.

[14] O'Byrne PM, Jenkins C, Bateman ED (2017). The paradoxes of asthma management: time for a new approach?. Eur Respir J.

[15] Mahboub BHSH, Santhakumar S, Soriano JB, Pawankar R (2010). Asthma insights and reality in the United Arab Emirates. Ann Thorac Med.

[16] Amin S, Soliman M, McIvor A, Cave A, Cabrera C (2020). Usage patterns of short-acting β_2_-agonists and inhaled corticosteroids in asthma: a targeted literature review. J Allergy Clin Immunol Pract.

[17] Bloom CI, Cabrera C, Arnetorp S, Coulton K, Nan C, van der Valk RJP (2020). Asthma-related health outcomes associated with short-acting β_2_-agonist inhaler use: an observational UK study as part of the SABINA global program. Adv Ther.

[18] Nwaru BI, Ekström M, Hasvold P, Wiklund F, Telg G, Janson C (2020). Overuse of short-acting β_2_-agonists in asthma is associated with increased risk of exacerbation and mortality: a nationwide cohort study of the global SABINA programme. Eur Respir J.

[19] Kaplan A, Mitchell PD, Cave AJ, Gagnon R, Foran V, Ellis AK (2020). Effective asthma management: is it time to let the air out of SABA?. J Clin Med.

[20] Global Initiative for Asthma (GINA). Global strategy for asthma management and prevention, 2019. https://ginasthma.org/gina-reports/. Accessed 22 Dec 2021.

[21] Baddar S, Baddar S, Rawas OA (2009). Oman National Asthma Guidelines/ Appendices Section. Guidelines for the Management of Asthma.

[22] Noibi S, Mohy A, Gouhar R, Shaker F, Lukic T, Al-Jahdali H (2020). Asthma control factors in the Gulf Cooperation Council (GCC) countries and the effectiveness of ICS/LABA fixed dose combinations: a dual rapid literature review. BMC Public Health.

[23] Cabrera CS, Nan C, Lindarck N, Beekman MJHI, Arnetorp S, van der Valk RJP (2020). SABINA: global programme to evaluate prescriptions and clinical outcomes related to short-acting β_2_-agonist use in asthma. Eur Respir J.

[24] Bateman ED, Price DB, Wang H-C, Khattab A, Schonffeldt P, Catanzariti A, et al. Short-acting β_2_-agonist prescriptions are associated with poor clinical outcomes of asthma: the multi-country, cross-sectional SABINA III study. Eur Respir J. 2022;59:2101402. 10.1183/13993003.01402-2021 PMC9068976 34561293

[25] Janson C, Menzies-Gow A, Nan C, Nuevo J, Papi A, Quint JK (2020). SABINA: an overview of short-acting β_2_-agonist use in asthma in European countries. Adv Ther.

[26] Reddel HK, Taylor DR, Bateman ED, Boulet LP, Boushey HA, Busse WW (2009). An official American Thoracic Society/European Respiratory Society statement: asthma control and exacerbations: standardizing endpoints for clinical asthma trials and clinical practice. Am J Respir Crit Care Med.

[27] Abuzakouk M, Jacob S, Ghorab O (2020). Are the Global Initiative for Asthma (GINA) guidelines being correctly used to diagnose severe asthma in the UAE?. Cureus.

[28] AlNohair S (2014). Obesity in gulf countries. Int J Health Sci (Qassim).

[29] Balhareth A, Meertens R, Kremers S, Sleddens E (2019). Overweight and obesity among adults in the Gulf States: A systematic literature review of correlates of weight, weight-related behaviours, and interventions. Obes Rev.

[30] Alzaabi A, Al-Kaabi J, Al-Maskari F, Farhood AF, Ahmed LA (2019). Prevalence of diabetes and cardio-etabolic risk factors in young men in the United Arab Emirates: A cross-sectional national survey. Endocrinol Diabetes Metab.

[31] Hirose M, Horiguchi T (2017). Asthma phenotypes. J Gen Fam Med.

[32] Alzaabi A, Idrees M, Behbehani N, Salah F (2021). Patients' and physicians' attitudes and perception about asthma in the Gulf: a subset analysis from the asthma insights and management survey in the Gulf and Russia. Allergy Asthma Proc.

[33] Reddel HK, Ampon RD, Sawyer SM, Peters MJ (2017). Risks associated with managing asthma without a preventer: urgent healthcare, poor asthma control and over-the-counter reliever use in a cross-sectional population survey. BMJ Open.

[34] FitzGerald JM, Tavakoli H, Lynd LD, Al Efraij K, Sadatsafavi M (2017). The impact of inappropriate use of short acting beta agonists in asthma. Respir Med.

[35] Abdo-Rabbo A, Al-Ansari M, Gunn BC, Suleiman BJ (2009). The use of medicines in oman: public knowledge, attitudes and practices. Sultan Qaboos Univ Med J.

[36] Abahussain EA, Ball DE, Matowe WC (2006). Practice and opinion towards disposal of unused medication in Kuwait. Med Princ Pract.

[37] Dubai Health Authority. NABIDH. https://nabidh.ae/#/comm/about. Accessed 22 Dec 2021.

[38] Fahmy SA, Abu-Gharbieh E, Hamidi S (2016). Patterns of prescribing and utilization of asthma medications in a tertiary hospital in Dubai, United Arab Emirates. Trop J Pharm Res.

[39] Al-Mahrezi A, Baddar S, Al-Siyabi S, Al-Kindi S, Al-Zakwani I, Al-Rawas O (2018). Asthma clinics in primary healthcare centres in Oman: do they make a difference?. Sultan Qaboos Univ Med J.

[40] Alzaabi A, Idrees M, Behbehani N, Khaitov MR, Tunceli K, Urdaneta E (2018). Cross-sectional study on Asthma Insights and Management in the Gulf and Russia. Allergy Asthma Proc.

[41] Kosoy I, Lew E, Ledanois O, Derrickson W (2022). Characterization of uncontrolled, severe asthma patients with type 2 inflammation (T2): results from a physician survey across countries from Latin American, Eurasian Middle East regions and China. J Asthma.

[42] Sullivan PW, Ghushchyan VH, Globe G, Schatz M (2018). Oral corticosteroid exposure and adverse effects in asthmatic patients. J Allergy Clin Immunol.

[43] Sá-Sousa A, Almeida R, Vicente R, Nascimento N, Martins H, Freitas A (2019). High oral corticosteroid exposure and overuse of short-acting beta_2_-agonists were associated with insufficient prescribing of controller medication: a nationwide electronic prescribing and dispensing database analysis. Clin Transl Allergy.

[44] Bloechliger M, Reinau D, Spoendlin J, Chang SC, Kuhlbusch K, Heaney LG (2018). Adverse events profile of oral corticosteroids among asthma patients in the UK: cohort study with a nested case-control analysis. Respir Res.

[45] Waljee AK, Rogers MA, Lin P, Singal AG, Stein JD, Marks RM (2017). Short term use of oral corticosteroids and related harms among adults in the United States: population based cohort study. BMJ.

[46] Bourdin A, Adcock I, Berger P, Bonniaud P, Chanson P, Chenivesse C, et al. How can we minimise the use of regular oral corticosteroids in asthma? Eur Respir Rev. 2020;29:190085. 10.1183/16000617.0085-2019 PMC9488989 32024721

[47] Global Initiative for Asthma (GINA). Global strategy for asthma management and prevention, 2022. https://ginasthma.org/gina-reports/. Accessed 26 May 2022.

[48] Abduelkarem AR, Othman AM, Abuelkhair ZM, Ghazal MM, Alzouobi SB, El Zowalaty ME (2019). Prevalence of self-medication with antibiotics among residents in United Arab Emirates. Infect Drug Resist.

[49] Braido F (2013). Failure in asthma control: reasons and consequences. Scientifica.

[50] Khadadah M, Mahboub B, Al-Busaidi NH, Sliman N, Soriano JB, Bahous J (2009). Asthma insights and reality in the Gulf and the near East. Int J Tuberc Lung Dis.

[51] Song WJ, Lee JH, Kang Y, Joung WJ, Chung KF (2019). Future risks in patients with severe asthma. Allergy Asthma Immunol Res.

[52] Sears MR (2019). Can we predict exacerbations of asthma?. Am J Respir Crit Care Med.

[53] Reddel HK, FitzGerald JM, Bateman ED, Bacharier LB, Becker A, Brusselle G (2019). GINA 2019: a fundamental change in asthma management: Treatment of asthma with short-acting bronchodilators alone is no longer recommended for adults and adolescents. Eur Respir J.

