# Supplementary material for: Over-prescription of short-acting β2-agonists and asthma management in the Gulf region: a multicountry observational study
Source: Asthma Res Pract. 2022 Jul 7;8:3. doi: 10.1186/s40733-022-00085-5 (PMC9260980; doi:10.1186/s40733-022-00085-5)
Supplement: Supplementary file 1 — Additional file 1: Supplementary Table 1. Study sites in SABINA Gulf study. [file 40733_2022_85_MOESM1_ESM.docx]

**Supplementary File**

**Over-prescription of short-acting β_2_-agonists and asthma management in the Gulf region: A multicountry observational study**

Ashraf Alzaabi,^1^ Nasser Al Busaidi,^2^ Rohit Pradhan,^1^ Fathelrahman Shandy,^3^ Naseem Ibrahim,^4^ Moulham Ashtar,^5^ Khaled Khudadah,^6^ Khaled Hegazy,^7^ Mohamed Samir,^8^ Mohamed Negm,^9^ Hisham Farouk,^10^ Arwa Al Khalidi,^10^ Maarten Beekman^11^

**Supplementary Table 1.** Study sites in SABINA Gulf study

| **Investigator name** | **Hospital/clinic Name** | **City, Country** | **Setting** |
| --- | --- | --- | --- |
| Nasser Al Busaidi | Royal Hospital | Muscat, Oman | Hospital, tertiary center |
| Khaled Khudadah | Ahmadi Hospital | Kuwait City, Kuwait | Primary healthcare clinic |
| Khaled Hegazy | Sabah El-Salem PHC | Kuwait City, Kuwait | Primary healthcare clinic |
| Mohamed Samir | Rumaitheya PHC | Kuwait City, Kuwait | Primary healthcare clinic |
| Mohamed Negm | Al Adan Hospital | Kuwait City, Kuwait | Hospital, tertiary center |
| Ashraf Alzaabi | Zayed Military Hospital | Abu Dhabi, UAE | Hospital, tertiary center |
| Rohit Pradhan | Zayed Military Hospital | Abu Dhabi, UAE | Hospital, tertiary center |
| Fathelrahman Shandy | Rashid Hospital, DHA | Dubai, UAE | Hospital, tertiary center |
| Naseem Ibrahim | Khalidiya MOH PHC | Sharjah, UAE | Primary healthcare clinic |
| Moulham Ashtar | Al Badaa PHC, DHA | Dubai, UAE | Primary healthcare clinic |
| Fayeza Alameri | Family Medicine Department, Zayed Military Hospital | Abu Dhabi, UAE | Primary healthcare clinic |
| Shatha Al Suwaidi | Al Mankhool PHC, DHA | Dubai, UAE | Primary healthcare clinic |
| Amal Al Zaabi  Leila Osman | AL Towar PHC, DHA | Dubai, UAE | Primary healthcare clinic |
| Hala Boushra | Al Riqqa MOH PHC | Sharjah, UAE | Primary healthcare clinic |
| Naim Hazzouri | Al Muhasinah MOH PHC | Dubai, UAE | Primary healthcare clinic |
| Aisha Salim  Safeya AlKaabi | Al Mamzar PHC, DHA | Dubai, UAE | Primary healthcare clinic |
